# Trends in adverse drug reaction reporting by dentists: a 15-year analysis of German pharmacovigilance data

**DOI:** 10.1007/s00784-026-06889-6

**Published:** 2026-04-27

**Authors:** Frank Halling, Rainer Lutz, Axel Meisgeier

**Affiliations:** Department of Oral and Craniomaxillofacial Surgery, UKGM GmbH, University Hospital Marburg and Faculty of Medicine, Philipps University, 35043 Marburg, Germany

**Keywords:** Adverse drug reaction, Antibiotic, Clindamycin, Dentistry, Pharmacovigilance, Underreporting

## Abstract

**Objectives:**

Dentists frequently encounter adverse drug reactions (ADRs) in their day-to-day work. Despite the legal obligation in Germany to report ADRs, systematic evaluations of dental pharmacovigilance data remain scarce. This study aimed to analyze ADR reports submitted by dentists to the Drug Commission of the German Dental Association.

**Materials and methods:**

All ADR reports submitted between 2010 and 2024 were analyzed according to year, medication class, and affected organ system. Incidence rates per dentist were calculated and trends over time were assessed using Poisson regression analysis.

**Results:**

A total of 983 ADR reports were included. Antibiotics accounted for the majority of reports (59.4%), followed by local anesthetics (15.7%). The most frequently affected organ systems were the skin (39.8%) and the gastrointestinal tract (21.7%). On average, 65.5 reports were submitted annually. The overall incidence rate was 91.7 reports per 100,000 dentist years, declining by 9.5% annually over the study period. Clindamycin had the highest incidence of ADR reports (26.7 per 100,000 dentist years) but also the steepest annual decrease (–17.1% per year).

**Conclusion:**

The proportion of ADRs reported by dentists is low compared with the expected number of ADRs, indicating substantial underreporting. A significant decline in reporting was observed over time. Consistent with previous studies, antibiotics and cutaneous reactions predominated. The reduction in clindamycin-related ADR reports reflects a substantial decrease in clindamycin prescriptions in Germany.

**Clinical relevance:**

Mandatory spontaneous reporting remains essential for dental pharmacovigilance. However, implementing digital tools and low-threshold reporting systems could improve the quantity and quality of future ADR reports.

**Supplementary Information:**

The online version contains supplementary material available at 10.1007/s00784-026-06889-6.

## Introduction

Adverse drug reactions (ADRs) have a significant impact on patients’ treatment in both medical and dental practice, as they may compromise patient safety, influence clinical outcomes, and contribute to escalating healthcare expenditures. The increasing complexity of pharmacological treatment concepts, an aging society, and the rising prevalence of multimorbidity signify that ADRs persist as a challenge in contemporary healthcare. According to Aronson and Ferner, an ADR is defined as a reaction that is appreciably harmful or unpleasant and that results from an intervention related to the use of a medicinal product. As stated in the literature, ADRs usually predict hazard from future administration and warrant prevention, or specific treatment, or alteration of the dosage regimen, or withdrawal of the product [1]. The prevalence of ADRs in dental practice has been documented to range from 5% to 10% among patients receiving dental care, indicating a considerable impact on treatment outcomes [2]. In contemporary dental practice, the utilization of pharmaceutical agents is limited to a small number of drug groups, including antibiotics, analgesics, and local anesthetics [2]. These agents have the potential to cause ADRs. The rising prevalence of multimorbidity in aging societies is accompanied by complex pharmacological treatment concepts that adhere to professional medical guidelines. In conjunction with age-related alterations in pharmacokinetics and pharmacodynamics, the prevalence of ADRs and drug-drug interactions among the elderly population is significantly higher compared to that observed in young adults [3–5]. Consequently, dental practitioners frequently encounter adverse effects resulting from medications they did not personally prescribe [6]. The management of these reactions poses challenges for dental practitioners, who must balance effective dental treatment with minimizing the risk of harm.

These adverse effects of pharmaceuticals may manifest in a variety of ways, including dermal or mucosal reactions, gastrointestinal disturbances, and systemic toxicity. However, unanticipated incidents within the oral and maxillofacial region, such as bisphosphonate-associated osteonecrosis of the jaw, underscore the importance of thorough spontaneous reporting of adverse events [7].

The detection, assessment, and prevention of ADRs is known as pharmacovigilance. Since 1968, the World Health Organization (WHO) has utilized an international pharmacovigilance program to monitor and examine the effects of pharmaceutical agents [8]. Pharmacovigilance is a critical component in ensuring the safety of medical products. In the contemporary era, a plethora of methodologies have been developed for the purpose of modern pharmacovigilance. These methodologies include, but are not limited to, spontaneous reporting databases, electronic health record monitoring and research frameworks, social media surveillance, and the utilization of digital devices [9]. The most important aspect of ensuring drug safety is the spontaneous reporting of suspected ADRs by various stakeholders, including physicians, dental practitioners, pharmacists, industry, and patients. A substantial body of research has identified underreporting of ADRs as a pervasive phenomenon within the health sector [6, 10–12]. A paucity of data exists concerning the knowledge, contributions, and perceptions of dentists regarding pharmacovigilance activities. This study aims to evaluate the state of pharmacovigilance by conducting a comprehensive analysis of all dental ADR reports submitted to the German Dental Association’s Drug Commission between 2010 and 2024.

## Materials & methods

Dentists in Germany are required by law to report adverse drug reactions (ADR). The obligation for dentists to report ADRs arises from the Dental Professional Code of Conduct (PCC; *“Musterberufsordnung”*). According to Section 2 (6) of the PCC by the German Dental Association (GDA; *“Bundeszahnärztekammer”*) dentists are obliged to report any adverse drug reactions that they become aware of during their dental practice to the Drug Commission of the German Dental Association (DCG; “*Arzneimittelkommision Zahnärzte*”). In particular, unexpected adverse drug reactions, adverse drug reactions to new drugs, and clinically particularly severe or prolonged courses should be reported.

The reports can be completed online on the website of the GDA or via postal delivery using a standardized registration form. This form is published in each edition of the official publication of the GDA (“*Zahnärztliche Mitteilungen*”). Each report is assigned an identification number. The patient identification details (initials, gender, date of birth) must be completed thoroughly to ensure the identification of duplicate reports of ADRs. All documents relevant to the reported ADR, particularly those pertaining to the symptoms and course of the event (e.g., examination findings, laboratory data), should be attached to the reporting form in copy. The medication believed to be the causative agent of the ADR must be explicitly specified, accompanied by data regarding other medications, including dosage and duration of application, as well as any known allergies. The treatment and outcome of the ADR (recovered, persistent, death) should be precisely provided. Should the dentist wish to receive a separate letter of advice for each reported ADR, a member of the DCG is available to prepare it. The completion of the report requires approximately 15 to 30 min. Upon receipt of the report (electronically or via postal mail), the reporter is duly issued a receipt of submission. All patient and physician-related data provided on the report form are treated confidentially in accordance with the provisions of the Federal Data Protection Act.

All ADR reports from the years 2010 to 2024 were provided by the DCG and analyzed according to year, medication class (e.g. antibiotic, local anesthetic, analgesic and others) and affected organ system (e.g. skin, gastrointestinal system, cardiovascular system, central nervous system, other organ system). The aggregated dataset provided by the DCG contained the following variables for each reporting year: total number of ADR reports, distribution by medication class (antibiotics, local anesthetics, analgesics, other), and distribution by primarily affected organ system (skin, gastrointestinal, CNS, cardiovascular, other). Individual case-level data (e.g., report IDs, patient demographics, or detailed drug names) were not available in the aggregated dataset, with the exception of antibiotic-specific data. Due to the retrospective nature of the study and the fully anonymous, aggregated data, the Research Ethics Committee of the Medical Faculty of the Philipps-Universität Marburg waived the need to obtain informed consent. In some cases, the reporting dentists list several drugs as possible causes of an observed ADR within a single report. The basis of the following statistical analysis is the number of ADR reports per year (RPY), rather than the number of ADRs itself. The normality of the distribution of continuous variables was tested by Kolmogorov-Smirnov test. Continuous variables with a normal distribution were presented as the mean and standard deviation. The means of 2 continuous normally distributed variables were compared by using an independent samples Student’s t-test. Differences were considered significant at a p-value of less than 0.05. In addition, adjusted rates of ADR reports per 100,000 dentist years were calculated using data on active dentists, which was also provided by the GDA, and reported with a 95% confidence interval. To avoid seasonal influences, the reports were analyzed on an annual basis. Incidence rates of reported ADRs were computed for each calendar year. The calculation of the 95% confidence intervals (95% CI) for the standardized incidences was performed using the delta method. Separate Poisson regression models were computed to investigate time trends, fitted with incidence rates of reported ADRs as dependent variables and medication class, organ system and year of reported ADRs starting from baseline year 2010 as independent variables. All models were adapted by descale adjustment to account for overdispersion of the outcome variable. Statistical analysis was performed using IBM SPSS Statistics Version 29.0 (IBM Deutschland GmbH, Böblingen, Germany).

## Results

**Fig. 1:**
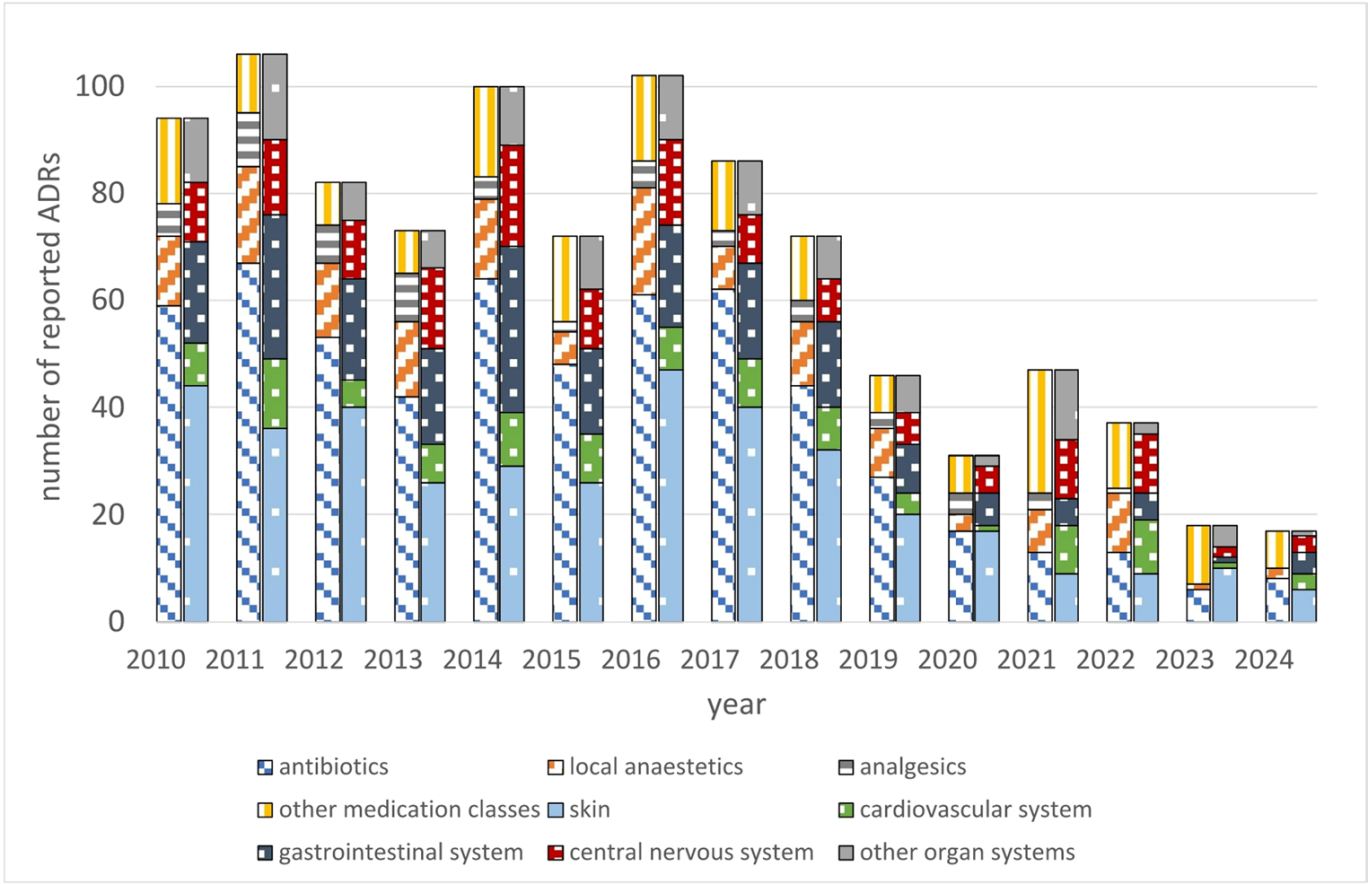
Number of ADR reports per year classified by medication class (left) and affected organ system (right)

During the observational period from 2010 to 2024, a total of 983 ADR reports were referred to the DCG and could be included in the study. The distribution by year, medication class and organ system is presented in Fig. 1 (see also Supplementary Table 1). The majority of reports concerned antibiotics (59.4%), which are one of the leading medication classes in dentistry and skin conditions (39.8%), which were the most affected organ system, followed by local anesthetics (15.7%) and gastrointestinal conditions (21.7%), respectively. The mean number of reports (65.5 ± 30.6/year) decreased by 57.2% during the observational period, falling from 89.4 RPY between 2010 and 2017 to 38.3 RPY between 2018 and 2024 (p-value < 0.05).

**Fig. 2:**
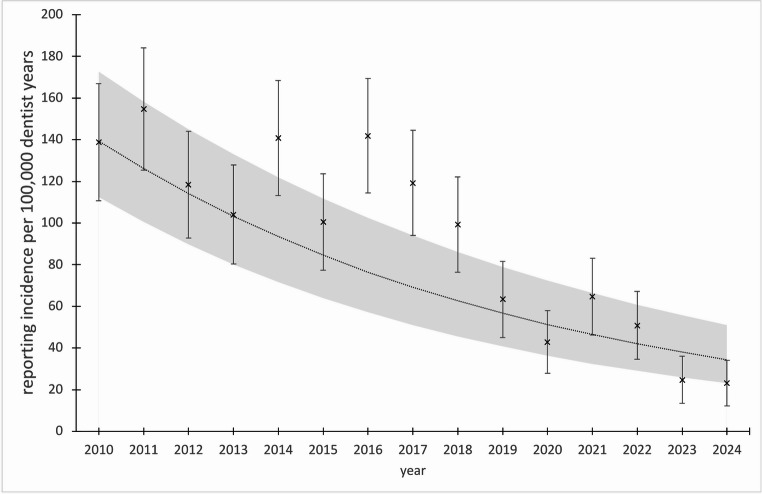
Incidence of ADR reports in Germany 2010—2024. x: incidence rates (95% confidence interval) per 100,000 dentist years per year. Dotted line: fitted Poisson regression model with 95% confidence Interval (grey area)

Table 1; Fig. 2 show the incidence rate of ADR reports in relation to the number of practicing dentists (see also Supplementary Tables 2 and 3). During the observational period (2010–2024) the incidence rate of ADRs reports was 91.7 per 100,000 dentist years [95% CI: 86.0-97.5] ranging from 154.7 [125.3–184.2] in 2011 to 23.1 [12.1–34.1] in 2024, a decrease of -85.1% within the observational period. The relative risk of 0.41 [0.35–0.48] indicates a significant reduction in reporting incidence of ADRs from 2018 to 2024 compared to 2010 to 2017.

Of the various medication classes, antibiotics accounted for the majority of reports, with an incidence rate of 56.6 [52.1–61.1] per 100,000 dentist years (Supplementary Table 2). This was followed by local anesthetics with an incidence rate of 14.9 [12.6–17.2] and analgesics with an incidence rate of 6.0 [4.5–7.4]. The highest antibiotic-related incidence was documented in 2011, at a rate of 97.8 [74.4–121.2], while the lowest rate was recorded in 2023 at a rate of 8.2 [1.6–14.8]. The relative risk of 0.36 [0.30–0.41] indicates a significant reduction in the reporting incidence regarding antibiotics from 2018 to 2024 compared to 2010 to 2017. A similar significant reduction in reporting incidence was observed for local anesthetics and analgesics from 2018 to 2024 compared to 2010 to 2017, with relative risks of 0.53 [0.38–0.74] and 0.43 [0.25–0.74] respectively. The incidence of reports on other medications is 17.6 [15.1–20.1] per 100,000 dentist years during the review period. The relative risk of 0.88 [0.66–1.17] for the observation period from 2018 to 2024 compared to 2010 to 2017 indicates a stable incidence of these reports (Table 1).

**Table 1:** Incidence of ADR reports and results of the Poisson regression models for all reports and stratified by medication class and affected organs system in Germany 2010—2024. Incidence rates (95% confidence interval) per 100,000 dentist years. Relative risk of the incidence of ADR reports (95% Confidence interval)

Years	Reporting incidence	Medication class				Organ system				
	Total	Antibiotics	Local anesthetics	Analgesics	Other medication classes	Skin	Cardio-vascular system	Gastro-intestinal system	Central nervous system	Other organ system
All years	91.7(86–97.5)	56.6 (52.1–61.1)	14.9 (12.6–17.2)	6.0 (4.5–7.4)	17.6 (15.1–20.1)	37.8 (34.1–41.4)	10.1 (8.2–12)	20.7 (18–23.4)	14.6 (12.3–16.9)	11.8 (9.7–13.9)
2010–2017	127.2 (117.8–136.1)	81.1 (73.7–88.2)	19.2 (15.6–22.5)	8.2 (5.8–10.2)	18.7 (15.1–21.9)	51.2 (45.3–56.8)	12.3 (9.4–14.8)	29.7 (25.2–33.8)	18.9 (15.3–22.1)	15.1 (11.9–18)
2018–2024	52.6 (46.3–58.5)	29.6 (24.8–33.9)	10.1 (7.4–12.5)	3.5 (1.9–4.7)	16.4 (12.9–19.5)	22.9 (18.8–26.7)	7.8 (5.3–9.8)	10.8 (7.9–13.2)	10 (7.2–12.3)	8.1 (5.7–10.2)
Relative Risk	0.41 (0.35–0.48)*	0.36 (0.30–0.44)*	0.53 (0.38–0.74)*	0.43 (0.25–0.74)*	0.88 (0.66–1.17)	0.45 (0.36–0.55)*	0.63 (0.43–0.93)*	0.36 (0.27–0.49)*	0.53 (0.38–0.74)*	0.54 (0.37–0.78)*
Annual Relative Risk	**0.905** (0.893–0.917)*	0.883 (0.868–0.898)*	0.908 (0.879–0.938)*	0.869 (0.824–0.916)*	0.985 (0.958–1.014)	0.899 (0.880–0.917)*	0.939 (0.902–0.974)*	0.875 (0.851–0.901)*	0.925 (0.896–0.956)*	0.914 (0.882–0.948)*

* *p*-value < 0.05

Of the organ system affected by the reported ADRs, the skin was the most affected organ system with an incidence of 37.8 [34.1–41.4] per 100,000 dentist years followed by the gastrointestinal system with an incidence of 20.7 [18.0–23.4], the central nervous system with an incidence rate of 14.6 [12.3–16.9] and the cardiovascular system with an incidence rate of 10.1 [8.2–12.0]. With regard to all organ systems involved, there was a significant decrease in the incidence of reports of adverse drug reactions from 2018 to 2024 compared to the period from 2010 to 2017 (p-value < 0.05) (Table 1, Supplementary Table 3).

**Fig. 3:**
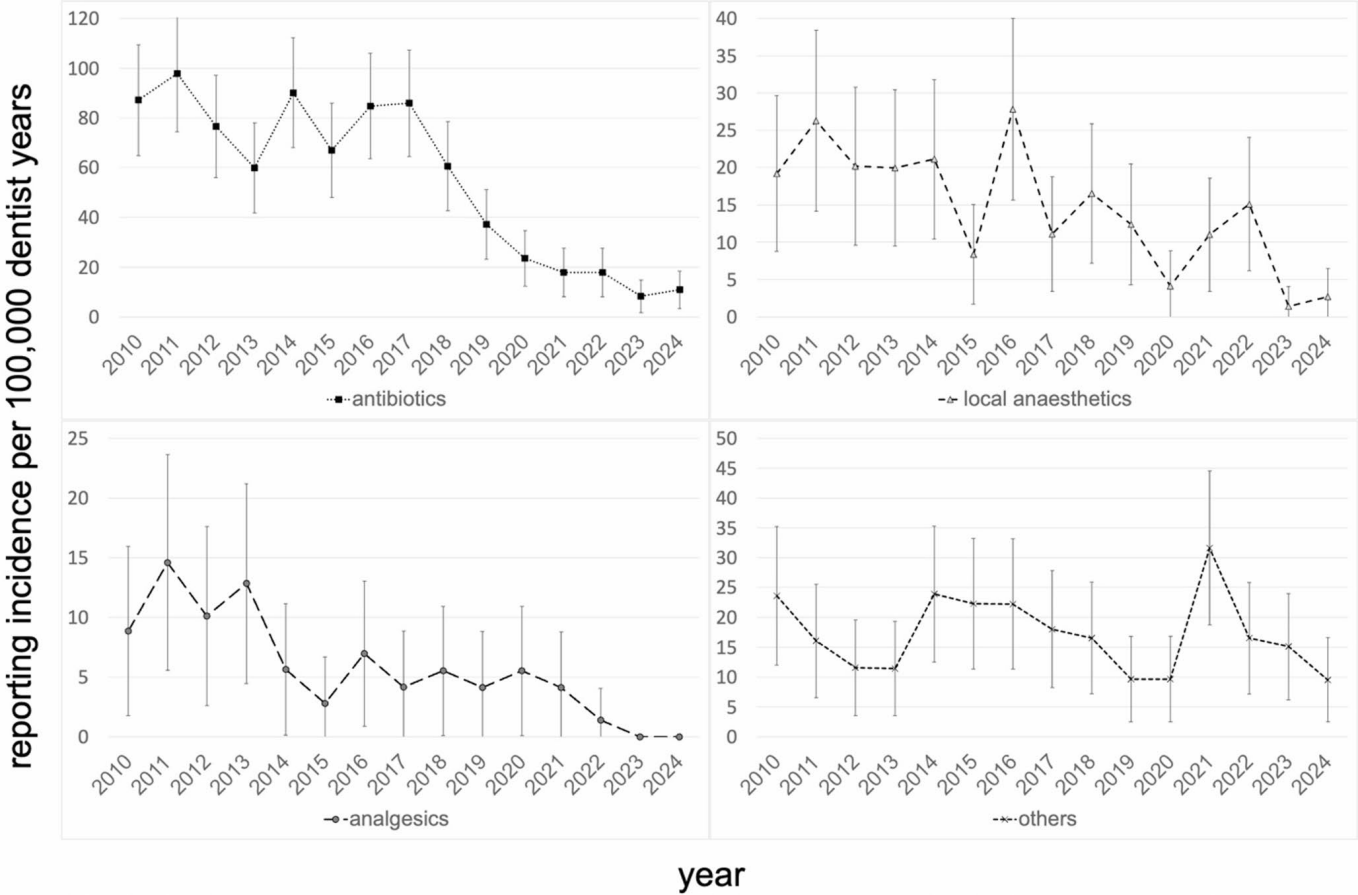
Incidence of ADR reports in Germany 2010—2024 by medication class. Incidence rates (95% confidence interval) per 100,000 dentist years

**Fig. 4:**
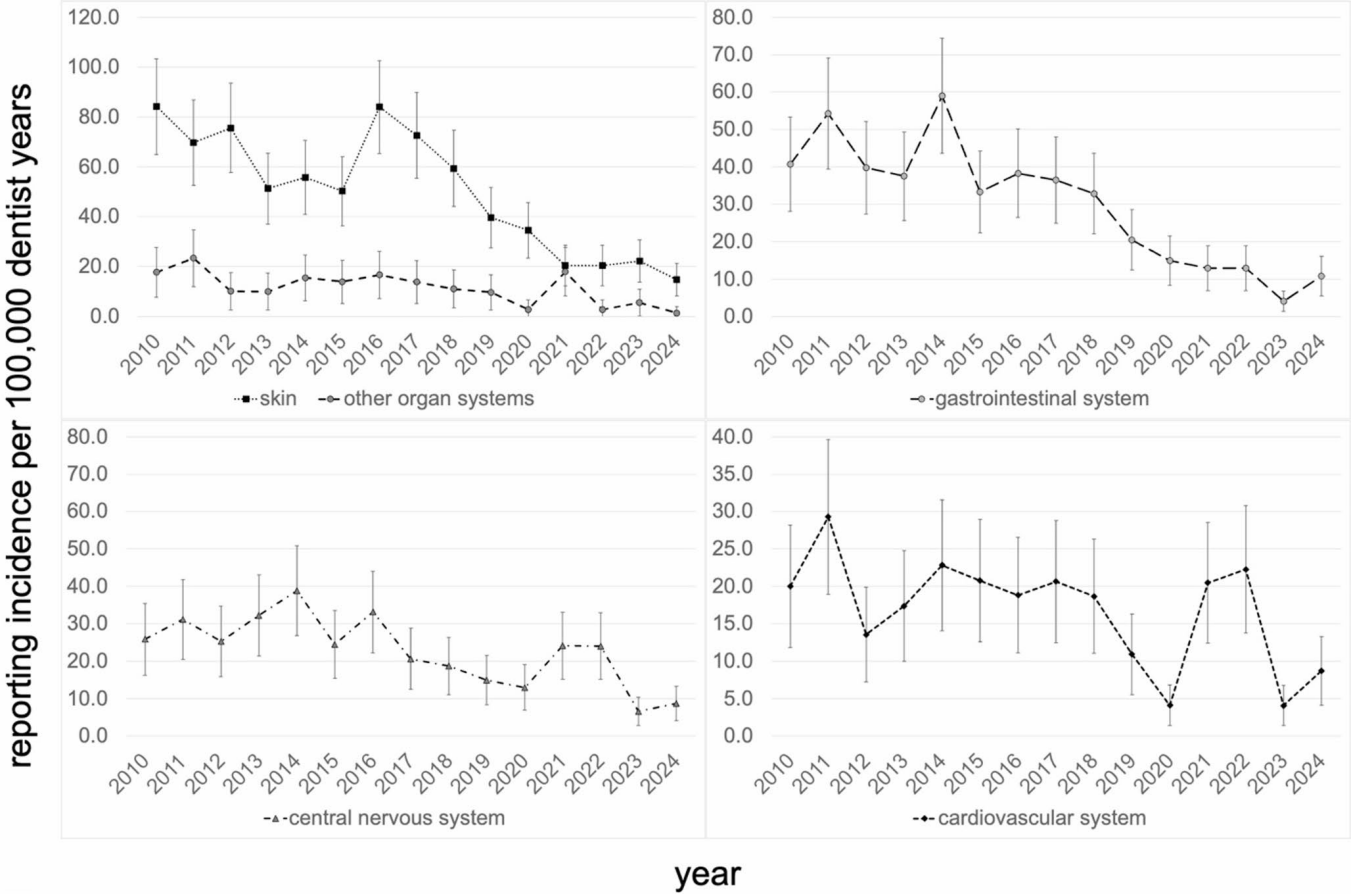
Incidence of ADR reports in Germany 2010—2024 by affected organ system. Incidence rates (95% confidence interval) per 100,000 dentist years

**Fig. 5:**
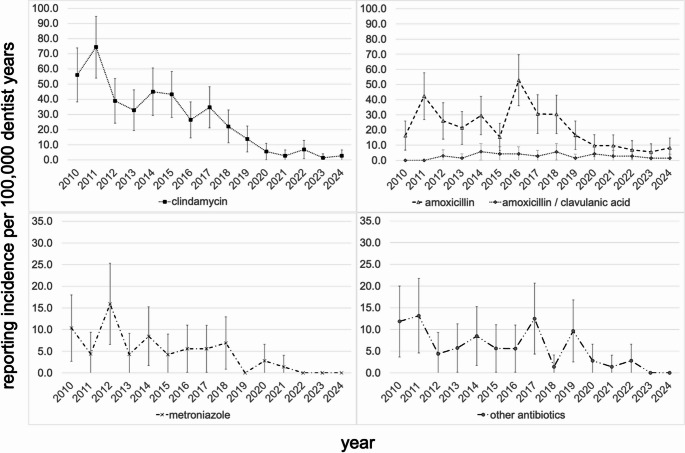
Incidence of ADR reports in Germany 2010—2024 regarding different antibiotics. Incidence rates (95% confidence interval) per 100,000 dentist years

The results of the time trend of reporting incidence from the adjusted Poisson models are shown in Table 1. During the observational period, we observed a significant annual percentage decrease in the incidence of ADR reports of -9.5% (relative risk per calendar year 0.905; 95% CI: 0.893–0.917, p-value < 0.001). The strongest decrease was seen in analgesics at -13.1% per year (RR: 0.869; 0.824–0.916; p-value < 0.001) and antibiotics at -11.7% per year (RR: 0.883; 0.868–0.898; p-value < 0.001) followed by local anesthetics at -9.2% per year (RR: 0.908; 0.879–0.938; p-value < 0.001). A slight, non-significant decrease was only seen in other medication classes (-1.5% per year, RR: 0.985; 0.958–1.014; p-value > 0.05). Of note is the reporting incidence regarding other medication classes of 31.6 in 2021, the year of the corona pandemic peak, which is 225% above the 2020 rate (9.7) and 92% above the 2022 rate (16.5) (Fig. 3 and Supplementary Table 2). With regard to the affected organ systems, the sharpest decline in the incidence of reports of ADRs was observed for gastrointestinal manifestations with an annual percentage decrease of -12.5% per year (RR: 0.875; 0.851–0.901; p-value < 0.001), followed by skin manifestations (-10.1% per year, RR: 0.899; 0.880–0.917; p-value < 0.001), followed by central nervous system affections (-7.5% per year, RR: 0.925; 0.896–0.956; p-value < 0.001) and cardiovascular manifestations (-6.1% per year, RR: 0.939; 0.902–0.974; p-value < 0.001). A significant decrease was also observed in the other organ systems (-8.6% per year, RR: 0.914; 0.882–0.948; p-value < 0.001; see Table 1). Furthermore, the reporting incidence regarding other organ systems of 17.9 during the peak of the corona pandemic in 2021 is striking being more than 530% higher than the rates in 2020 (2.8) and 2022 (2.7) (see Fig. 4 and Supplementary Table 3). Within the most reported medication class of antibiotics, clindamycin accounted for the highest number of reports, with an incidence rate of 26.7 [23.6–29.8] per 100,000 dentist years. This was followed by amoxicillin with an incidence rate of 21.3 [18.5–24.0] and metronidazole with an incidence rate of 4.6 [3.3–5.9] as shown in Table 2 (see also Supplementary Tables 4 and Fig. 5). The highest incidence rate related to clindamycin was documented in 2011, with a rate of 74.5 [54.0–94.9], while the lowest rate was recorded in 2023, with an incidence rate of 1.4 [0.0–4.1]. The relative risk of 0.18 [0.13–0.25] indicates a strong significant reduction in reporting incidence regarding clindamycin from 2018 to 2024 compared to 2010 to 2017. A significant reduction in reporting incidence was also observed for amoxicillin and metronidazole from 2018 to 2024 compared to 2010 to 2017, with relative risks of 0.42 [0.32–0.56] and 0.22 [0.10–0.46] respectively. On an annual basis the decrease was strongest for clindamycin (-17.1% per year, RR: 0.829; 0.807–0.851; p-value < 0.001), followed by metronidazole (-13.9% per year, RR: 0.861; 0.812–0.914; p-value < 0.001), followed by amoxicillin (-8.1% per year, RR: 0.919; 0.895–0.943; p-value < 0.001) (see Table 2).

**Table 2:** Incidence of ADR reports and results of the Poisson regression models for different antibiotics in Germany 2010—2024. Incidence rates (95% confidence interval) per 100,000 dentist years according to different antibiotics. Relative risk of the incidence of ADR reports (95% Confidence interval)

Years	Reporting incidence	Antibiotics				
	All antibiotics	Clindamycin	Amoxicillin	Amoxicillin/ Clavulanic acid	Metronidazole	Others
All years	56.6 (52.1–61.1)	26.7 (23.6–29.8)	21.3 (18.5–24)	2.7 (1.7–3.7)	4.6 (3.3–5.9)	5.6 (4.2–7)
2010–2017	81.1 (73.7–88.2)	43.8 (38.3–49.2)	29.3 (24.9–33.8)	2.7 (1.3–4)	7.3 (5.1–9.5)	8.4 (6–10.7)
2018–2024	29.6 (24.8–33.9)	7.9 (5.4–10.3)	12.4 (9.3–15.4)	2.7 (1.3–4.2)	1.6 (0.5–2.7)	2.6 (1.2–3.9)
Relative Risk	0.36 (0.3–0.44)*	0.18 (0.13–0.25)*	0.42 (0.32–0.56)*	1.03 (0.5–2.13)	0.22 (0.1–0.46)*	0.31 (0.17–0.56)*
Annual Relative Risk	0.883 (0.868–0.898)*	0.829 (0.807–0.851)*	0.919 (0.895–0.943)*	0.997 (0.930–1.070)	0.861 (0.812–0.914)*	0.886 (0.841–0.934)*

*p-value < 0.05

## Discussion

Spontaneous ADR reporting is a critical component of post-marketing drug surveillance, as extensive clinical trials have been known to fail to detect certain side effects [13, 14]. This approach is characterized by its economic efficiency, simplicity, and widespread use in identifying novel safety concerns associated with pharmaceuticals. Nevertheless, underreporting constitutes its primary constraint [11, 15]. Reports on ADRs need only four items of information for a valid report: an identifiable patient, a clinical reaction, a suspected medicinal product and an identifiable reporter [16]. In Germany, the proportion of reports to the Federal Institute for Drugs and Medical Devices (BfArM; *Bundesinstitut für Arzneimittel und Medizinprodukte*) originating from dental practitioners was found to be minimal at 0.15% [17]. This percentage is consistent with recently published French data. There a retrospective analysis was conducted over a ten-year period, during which all cases recorded by dentists in the French national pharmacovigilance database (BNPV) were extracted. Comparable to Germany these reports constitute only 0.06% of all cases that have been registered over a span of ten years [6].

The present study sought to analyze the reporting situation in Germany by performing a cumulative evaluation of spontaneous dental reports to the German Dental Association’s Drug Commission over a 15-year period. The mean number of ADR reports per year was 65.5. The findings of our analysis are comparable to data from France. Vagnet et al. found an average number of 51 dental reports of ADRs recorded between 2013 and 2023 in the French pharmacovigilance database [6]. A substantial decline in the incidence rates of ADR reports was observed during the observational period (-85.1%), with a notable annual percentage decrease of 9.5%. In comparison to these data, the total number of spontaneous reports per year to the German Federal Institute for Drugs and Medical Devices (BfArM) has exhibited a consistent upward trend between 2012 and 2019. However, a subsequent decline was observed in the subsequent period until 2024 [17]. In the United Kingdom, there has been a 37% decline in ADR reports from healthcare professionals over a 9-year period up to 2013 [18].

The underreporting of ADRs is a well-documented issue among healthcare professionals, including physicians, pharmacists, and dental professionals [4, 12, 19, 20]. A systematic review revealed a median underreporting rate of 94% across the 37 studies examined [11]. The majority of international studies regarding the awareness of ADRs in dentistry focused on the findings of domestic questionnaire surveys with a minimum of 98 and a maximum of 574 participating dental practitioners, e.g. in Albania [21], in England [19, 22], in India [12, 20, 23, 24] and Senegal [25]. The consensus of these surveys indicates that, in congruence with other health care professionals, dental practitioners perceive the importance of recognition of ADRs for patients’ safety, but there is substantial evidence of underreporting and a paucity of awareness regarding ADR reporting.

In Germany, up to now there exists no study investigating the attitude of dental practitioners towards ADR reporting. The data presented in this analysis reveal a continuous and dramatic decline over the observation period, particularly pronounced in the time period between 2018 and 2024. This phenomenon could be due to the fact that the last printed synopsis of ADR reports in Germany covered the data of 2016 and was published in 2018 [26]. Between 2018 and mid-2023 the ADR reports were not publicly accessible. Therefore, reporting of ADRs has lost its direct media presence for most of the German dentists. Beside this, the topic pharmacovigilance is rarely addressed in dental education activities or German-language dental journals. The importance of continuous education and training programs must be emphasized because recently published data show that more experienced practitioners are more likely to report ADRs [20]. In contrast, a current Cochrane review showed low-certainty evidence for a substantial increase of ADR reporting following education sessions, together with reminder cards and standardized discharge form with added ADR items compared to usual practice of spontaneous reporting. The evidence of other interventions, such as informational letters or emails and financial incentives, is uncertain [27].

A recent publication has provided a comprehensive analysis of the significant factors associated with underreporting of ADRs by healthcare professionals in the European Economic Area (EEA). The most prevalent attitudes that led to underreporting were as follows: 86.2% of respondents indicated that only serious ADRs need to be reported, 84.6% indicated lethargy (e.g., procrastination, lack of interest, and other excuses), 46.2% indicated complacency (i.e., the belief that only well-tolerated drugs are allowed on the market), 44.6% indicated diffidence (i.e., fear of appearing ridiculous for reporting merely suspected ADRs), and 33.8% indicated insecurity (i.e., it is nearly impossible to determine whether or not a drug is responsible for a specific adverse reaction) [10]. Notably, in comparison to patients, healthcare professionals reported serious ADRs with greater frequency and completeness than non-serious ADRs [28]. The drugs most commonly inducing ADRs in a dental setting are antibiotics, local anesthetics and nonsteroidal anti-inflammatory drugs (NSAID) [6, 20]. Awareness regarding ADRs induced by antibiotics is well-developed within the field of dentistry, as evidenced by numerous studies [6, 20, 23, 25]. The present study revealed that the majority of reports pertained to antibiotics (59.4%) with an incidence of 56.6 per 100,000 dentist years. These were followed by local anesthetics (14.9 per 100,000 dentist years) and analgesics (6.0 per 100,000 dentist years). The same sequence was identified in a French retrospective analysis of pharmacovigilance data [6]. During the investigation period, clindamycin was the medication most frequently reported, with an incidence rate of 26.7 per 100,000 dentist years. Alongside NSAIDs and antiepileptics, antibiotics were identified as the most prevalent pharmaceutical agents associated with cutaneous adverse effects [29]. As indicated in the existing literature, cutaneous reactions were the most frequently observed adverse drug reaction (ADR), with an incidence rate of 37.8 per 100,000 dentist years [6, 20]. This was followed by gastrointestinal side effects, with an incidence rate of 20.7. The distinct annual decline in ADR reports concerning gastrointestinal manifestations (− 12.5% per year) and skin manifestations (-10.1% per year) is clearly associated with the negative trend in ADR reports, particularly those concerning antibiotics (-11.7% per year). A meticulous examination reveals a significant and comparatively stronger annual decrease in reports concerning clindamycin (-17.1% per year) in comparison to amoxicillin (-8.1% per year). This trend for clindamycin appears to be consistent with the documented decline in clindamycin prescriptions in Germany between 2012 and 2024 [30, 31]. The effect could be elucidated by a series of recent publications that have adopted a critical stance on the prolonged utilization of clindamycin within the realm of German dentistry [30–32]. A recent series of studies has indicated that clindamycin demonstrates the highest incidence of adverse drug reactions (ADRs), particularly in terms of gastrointestinal disturbances, among the antibiotics commonly prescribed by dentists [30, 31, 33, 34].

In contrast to the increasing number of ibuprofen prescriptions between 2012 and 2021 in Germany [30], the total number of ADR reports concerning analgesics was extremely low (6.2% of all ADR reports) and decreased the most within the observational period (-13.1% per year). This effect may be explained by the usually short duration of analgesic treatment in dentistry and a low awareness of the analgesic-specific contraindications and side-effects [35]. NSAIDs should be used very cautiously above all in elderly patients with multimorbidity and those with renal and/or cardiovascular diseases [5]. Unlike other studies, the proportion of ADR reports concerning local anesthetics was relatively low (15.7%) [6, 36]. There was an annual reporting decrease of 9.2%. In this context, the results of a large observational study have demonstrated that the frequency of adverse reactions related to articaine, which dominates the German market [37], is low [38]. A multitude of studies have indicated that the incidence of adverse drug reactions (ADRs) associated with epinephrine-containing local anesthetics is considerably higher than that associated with epinephrine-free anesthetics [36, 39]. In the present study, an attempt was made to differentiate between reported ADRs to local anesthetics according to different epinephrine-containing formulations. Unfortunately, this was not possible due to the aggregated structure of the source data. Regarding the distribution of reports on the course of the COVID-19 pandemic there have been considerable fluctuations. The number of reports reached an all-time low in 2020. In 2021, which corresponded to the peak of the pandemic, there was a significant increase in the incidence of ADR reports. This increase contrasts with the overall trend in the preceding and subsequent years and is, beside local anesthetics, primarily related to other medications containing the SARS-CoV-2 vaccines. A similar effect related to the COVID-19 pandemic was documented by Vagnet et al. in the retrospective ten-year study who ascertained a proportion of 24.3% of all ADR reports in 2021 associated with the SARS-CoV-2 vaccines [6].

The study’s strengths and limitations must be discussed. The strength of this study lies in its comprehensive analysis of all reported cases of adverse drug reactions (ADRs) reported by German dentists over a 15-year period. This is the first study to deal with this topic based on German pharmacovigilance data. Most of existing studies on dental ADR reports have focused exclusively on the oral and oropharyngeal side effects of common drugs [40, 41]. The results of the study are limited due to its retrospective design and the restriction to one country. It is unclear whether these results can be generalized to ADR reports from other countries. However, this could be a topic worthy of further research. Furthermore, the aggregated database does not contain any information about the accuracy of the original ADR reports or even the number of inaccurate reports. Especially in patients with multimorbidity it can be difficult for dentists to distinguish ADRs from symptoms of underlying diseases. A new approach to improve ADR reporting are international campaigns like #MedSafetyWeek by the WHO. This campaign addresses medicines regulators, national pharmacovigilance centers, and international organizations around the world to encourage reporting of side effects to the relevant drug authorities. The implementation of digital tools and low threshold reporting systems may enhance both the quantity and quality of future ADR reports [42]. The integration of “big data” sources in conjunction with the application of machine learning algorithms for data analysis, has the potential to markedly enhance drug safety monitoring in the future. This domain remains both intriguing and largely unexplored, offering significant potential for research.

## Conclusion

Timely and voluntary ADR reporting plays a crucial role in mitigating the morbidity linked to unexpected reactions and improper medication usage. In this context, dentists, like other health care professionals, play a distinct role in safeguarding patients’ well-being. The substantial decrease in ADR reports submitted by dentists in Germany over the past fifteen years is indicative of a pervasive issue of underreporting within the healthcare sector as a whole. The significance of pharmacovigilance for patient safety should be addressed with utmost urgency on official dental websites, frequently read dental journals, and campaigns. Given the prevalence of adverse drug reactions (ADRs) and the potential for severe adverse effects, it is imperative that dentists prioritize the management of commonly prescribed medication groups, such as antibiotics and analgesics. Furthermore, it is imperative to streamline dental ADR reporting to the greatest extent possible through the utilization of digital tools and low-threshold reporting systems.

## Supplementary Information

Supplementary Material 1 (DOCX 33.3 KB)

## Data Availability

The datasets generated during and analyzed during the current study are available from the corresponding author on reasonable request.

## References

[1] Aronson JK, Ferner RE (2005) Clarification of terminology in drug safety. Drug Saf 28:851–870. 10.2165/00002018-200528100-00003 16180936 10.2165/00002018-200528100-00003

[2] Becker DE (2014) Adverse Drug Reactions in Dental Practice. Anesth Prog 61:26–34. 10.2344/0003-3006-61.1.26 24697823 10.2344/0003-3006-61.1.26 PMC3975611

[3] Hung A, Kim YH, Pavon JM (2024) Deprescribing in older adults with polypharmacy. BMJ. 10.1136/bmj-2023-074892 38719530 10.1136/bmj-2023-074892

[4] Lavan AH, Gallagher P (2015) Predicting risk of adverse drug reactions in older adults. Therapeutic Adv Drug Saf 7:11–22. 10.1177/2042098615615472 10.1177/2042098615615472 PMC4716390 26834959

[5] Ouanounou A, Haas DA (2014) Pharmacotherapy for the Elderly Dental Patient. J Can Dent Assoc 80:f18 26679331

[6] Vagnet A (2025) Analyse de la déclaration de pharmacovigilance par les chirurgiens-dentistes en France: bilan des 10 dernières années. Therapies 80:647–652. 10.1016/j.therap.2025.02.011 10.1016/j.therap.2025.02.011 40140295

[7] Carnelio S, Khan SA, Rodrigues G (2011) Pharmacovigilance in clinical dentistry: overlooked or axiomatic? Gen Dent 59:24–28 21613036

[8] World Health Organization (2025) Pharmacovigilance resources. World Health Org*.* https://www.who.int/teams/regulation-prequalification/regulation-and-safety/pharmacovigilance. Accessed 20 Dec 2025

[9] Lavertu A, Vora B, Giacomini KM, Altman R, Rensi SA (2021) New Era in Pharmacovigilance: Toward Real-World Data and Digital Monitoring. Clin Pharmacol Ther 109:1197–1202. 10.1002/cpt.2172 33492663 10.1002/cpt.2172 PMC8058244

[10] García-Abeijon P (2023) Factors Associated with Underreporting of Adverse Drug Reactions by Health Care Professionals: A Systematic Review Update. Drug Saf 46:625–636. 10.1007/s40264-023-01302-7 37277678 10.1007/s40264-023-01302-7 PMC10279571

[11] Hazell L, Shakir SAW (2006) Under-Reporting of Adverse Drug Reactions. Drug Saf 29:385–396. 10.2165/00002018-200629050-00003 16689555 10.2165/00002018-200629050-00003

[12] Yadav L (2024) Assessment of Knowledge, Attitude, and Practice of Pharmacovigilance and Materiovigilance among Oral Health Practitioners in India. J Pharm Bioallied Sci 16:S202–S205. 10.4103/jpbs.jpbs_456_23 38595538 10.4103/jpbs.jpbs_456_23 PMC11001093

[13] Amery WK (1999) Why there is a need for pharmacovigilance. Pharmacoepidemiol Drug Saf 8:61–64. 10.1002/(SICI)1099-1557(199901/02)8:1 15073950 10.1002/(SICI)1099-1557(199901/02)8:1<61::AID-PDS395>3.0.CO;2-A

[14] Huang YL, Moon J, Segal JBA (2014) Comparison of Active Adverse Event Surveillance Systems Worldwide. Drug Saf 37:581–596. 10.1007/s40264-014-0194-3 25022829 10.1007/s40264-014-0194-3 PMC4134479

[15] Montané E, Santesmases J (2020) Reacciones adversas a medicamentos. Medicina Clínica 154:178–184. 10.1016/j.medcli.2019.08.007 31771857 10.1016/j.medcli.2019.08.007

[16] Coleman JJ, Pontefract SK (2016) Adverse drug reactions. Clin Med 16:481–485. 10.7861/clinmedicine.16-5-481 10.7861/clinmedicine.16-5-481 PMC6297296 27697815

[17] Federal Institute for Drugs and Medical Devices (2024) Sachstandsberichte Nebenwirkungen 2024. Federal Institute for Drugs and Medical Devices. https://www.bfarm.de/SharedDocs/Downloads/DE/Arzneimittel/Pharmakovigilanz/Gremien/RoutinesitzungPar63AMG/96Sitzung/pkt-2-1-a.pdf?__blob=publicationFile. Accessed 20 Dec 2025

[18] Wise J (2013) GPs are urged to report adverse drug reactions after a 37% slump over nine years. BMJ 346:f690–f690. 10.1136/bmj.f690 23377613 10.1136/bmj.f690

[19] Yip J, Radford DR, Brown D (2013) How do UK dentists deal with adverse drug reaction reporting? Br Dent J 214:E22–E22. 10.1038/sj.bdj.2013.426 23619888 10.1038/sj.bdj.2013.426

[20] Rastogi I (2024) Challenges and opportunities in dentistry regarding adverse drug reactions. J Oral Med Oral Surg Oral Pathol Oral Radiol 10:185–190. 10.18231/j.jooo.2024.035

[21] Hoxha M, Spahiu E, Spahiu M, Zappacosta B (2024) Reporting of Adverse Drug Reactions by Dentists and Dental Patients in Albania. Int Dent J 74:242–245. 10.1016/j.identj.2023.08.005 37735045 10.1016/j.identj.2023.08.005 PMC10988243

[22] Patel MM, Radford DR, Brown D (2014) Preaching to the converted – optimising adverse drug reaction reporting by dentists. Br Dent J 217:E4–E4. 10.1038/sj.bdj.2014.598 25060479 10.1038/sj.bdj.2014.598

[23] Chari DN (2021) Adverse Drug Reaction. Adv Hum Biology 11:181–187. 10.4103/aihb.aihb_107_20

[24] Khan S, Goyal C, Tonpay SD (2015) A study of knowledge, attitudes, and practice of dental doctors about adverse drug reaction reporting in a teaching hospital in India. Perspect Clin Res 6. 10.4103/2229-3485.159938 10.4103/2229-3485.159938 PMC4504056 26229750

[25] Diouf M (2013) [Pharmacovigilance among dentists: a survey of practitioners in Dakar, Senegal]. Sante Publique 25:69–76 23705337

[26] Schindler C, Nagaba J, Stahlmann R (2018) [Adverse drug reaction reports for clindamycin decline for the first time]. ZM 108(9):970–976

[27] Shalviri G (2024) Improving adverse drug event reporting by healthcare professionals. Improving adverse drug event reporting by healthcare professionals. Cochrane Database Syst Rev 2024. 10.1002/14651858.CD012594.pub2 10.1002/14651858.CD012594.pub2 PMC11520514 39470185

[28] Dubrall D, Christ P, Domgörgen S, Schmid M, Sachs B (2025) Factors associated with the completeness of information provided in adverse drug reaction reports of physicians, pharmacists and consumers from Germany. Sci Rep 15. 10.1038/s41598-025-07973-9 10.1038/s41598-025-07973-9 PMC12229551 40610517

[29] Del Pozzo-Magana BR, Liy-Wong C (2024) Drugs and the skin: A concise review of cutaneous adverse drug reactions. Br J Clin Pharmacol 90:1838–1855. 10.1111/bcp.15490 35974692 10.1111/bcp.15490

[30] Albrecht H, Schiegnitz E, Halling F (2024) Facts and trends in dental antibiotic and analgesic prescriptions in Germany, 2012–2021. Clin Oral Invest 28. 10.1007/s00784-024-05497-6 10.1007/s00784-024-05497-6 PMC10794513 38231453

[31] Cirkel LL, Herrmann JM, Ringel C, Wöstmann B, Kostev K (2025) Antibiotic Prescription in Dentistry: Trends, Patient Demographics, and Drug Preferences in Germany. Antibiotics 14. 10.3390/antibiotics14070676 10.3390/antibiotics14070676 PMC12291895 40723979

[32] Gradl G, Kieble M, Nagaba J, Schulz M (2022) Assessment of the Prescriptions of Systemic Antibiotics in Primary Dental Care in Germany from 2017 to 2021: A Longitudinal Drug Utilization Study. Antibiotics 11. 10.3390/antibiotics11121723 10.3390/antibiotics11121723 PMC9774256 36551380

[33] Thornhill MH, Dayer MJ, Durkin MJ, Lockhart PB, Baddour LM (2019) Risk of Adverse Reactions to Oral Antibiotics Prescribed by Dentists. J Dent Res 98:1081–1087. 10.1177/0022034519863645 31314998 10.1177/0022034519863645 PMC8256247

[34] Geller AI (2018) National Estimates of Emergency Department Visits for Antibiotic Adverse Events Among Adults—United States, 2011–2015. J Gen Intern Med 33:1060–1068. 10.1007/s11606-018-4430-x 29679226 10.1007/s11606-018-4430-x PMC6025673

[35] Heimes D (2025) Dental recommendation and prescribing patterns for systemic analgesics - a cross-sectional study. Clin Oral Investig 29:383. 10.1007/s00784-025-06403-4 40658221 10.1007/s00784-025-06403-4 PMC12259747

[36] Bayram F, Akici A, Apari AM, Aydin V (2025) Analysis of Adverse Events Associated With Dental Local Anaesthetics Using Food and Drug Administration Adverse Event Reporting System Data. Int Dent J 75:1705–1712. 10.1016/j.identj.2025.03.002 40168927 10.1016/j.identj.2025.03.002 PMC11999206

[37] Halling F, Neff A, Ziebart T (2021) Local Anesthetic Usage Among Dentists: German and International Data. Anesth Prog 68:19–25. 10.2344/anpr-67-03-12 33827123 10.2344/anpr-67-03-12 PMC8033583

[38] Yamashita IC (2020) Observational study of adverse reactions related to articaine and lidocaine. Oral Maxillofacial Surg 24:327–332. 10.1007/s10006-020-00866-3 10.1007/s10006-020-00866-3 32524211

[39] Liu W, Yang X, Li C, Mo A (2013) Adverse drug reactions to local anesthetics: a systematic review. Oral Surg Oral Med Oral Pathol Oral Radiol 115:319–327. 10.1016/j.oooo.2012.04.024 22959146 10.1016/j.oooo.2012.04.024

[40] Ellefsen BS, Larsen KR, Reibel J, Kragelund C (2023) Danish post-marketing pharmacosurveillance of spontaneous Oral Adverse Drug Reactions 2009–2019. Oral Dis 30:1573–1582. 10.1111/odi.14559 36895115 10.1111/odi.14559

[41] Sportiello L (2024) Oropharyngeal Adverse Events to Drugs and Vaccines: Pharmacovigilance Data From Italy (2019–2021). Oral Dis 31:993–1005. 10.1111/odi.15145 39370676 10.1111/odi.15145 PMC12021317

[42] La Mantia G, Buttacavoli F, Panzarella V, Colella G, Capuano A, Sportiello L, Parrinello G, Morreale I, Oteri G, Bellavia G et al (2023) Oro-Dental Pharmacovigilance in the Digital Age: Promoting Knowledge, Awareness, and Practice in Italy through a Smart Combined System—A Conference at the 30th National Congress of the Italian College of University Professors of Dental Disciplines. Oral 3(3):411–419. 10.3390/oral3030033

