# Supplementary material for: Trends in adverse drug reaction reporting by dentists: a 15-year analysis of German pharmacovigilance data
Source: Clin Oral Investig. 2026 Apr 27;30(5):204. doi: 10.1007/s00784-026-06889-6 (PMC13121399; doi:10.1007/s00784-026-06889-6)
Supplement: Supplementary file 1 — Supplementary Material 1 (DOCX 33.3 KB) [file 784_2026_6889_MOESM1_ESM.docx]

**Supplementary Information File for:**

Trends in adverse drug reaction reporting by dentists: a 15-year analysis of German pharmacovigilance data

Frank Halling^1^, Rainer Lutz^1^, Axel Meisgeier^1*^

1. Department of Oral and Craniomaxillofacial Surgery, UKGM GmbH, University Hospital Marburg and Faculty of Medicine, Philipps University, Marburg, Germany

Corresponding author*

Priv.-Doz. Dr. Dr. Axel Meisgeier

Department of Oral and Craniomaxillofacial Surgery

UKGM GmbH, University Hospital Marburg

Faculty of Medicine, Philipps University, Marburg

35043 Marburg, Germany

e-mail: axel.meisgeier@med.uni-marburg.de

phone: +49 (0)6421 58-63237

**This document includes Supplementary Table 1, Supplementary Table 2, Supplementary Table 3 and Supplementary Table 4.**

**Supplementary Table 1:** Numbers of ADR reports in Germany 2010 – 2024 in total, according to medication class and according to affected organ system.

| **Years** | **Total ADRs** | **Medication class** |  |  |  |  | **Organ system** |  |  |  |  |
| --- | --- | --- | --- | --- | --- | --- | --- | --- | --- | --- | --- |
|  |  | **Antibiotics** | **Local anesthetics** | **Analgesics** | **Other medication classes** |  | **Skin** | **Cardio-vascular system** | **Gastro-intestinal system** | **Central nervous system** | **Other organ system** |
| 2010  2011  2012  2013  2014  2015  2016  2017  2018  2019  2020  2021  2022  2023  2024 | **94**  **106**  **82**  **73**  **100**  **72**  **102**  **86**  **72**  **46**  **31**  **47**  **37**  **18**  **17** | 59  67  53  42  64  48  61  62  44  27  17  13  13  6  8 | 13  18  14  14  15  6  20  8  12  9  3  8  11  1  2 | 6  10  7  9  4  2  5  3  4  3  4  3  1  0  0 | 16  11  8  8  17  16  16  13  12  7  7  23  12  11  7 |  | 44  36  40  26  29  26  47  40  32  20  17  9  9  10  6 | 8  13  5  7  10  9  8  9  8  4  1  9  10  1  3 | 19  27  19  18  31  16  19  18  16  9  6  5  5  1  4 | 11  14  11  15  19  11  16  9  8  6  5  11  11  2  3 | 12  16  7  7  11  10  12  10  8  7  2  13  2  4  1 |
| **Total**  Mean  SD | **983**  65.5  30.6 | **584**  38.9  22.6 | **154**  10.3  5.7 | **61**  4.1  3.0 | **184**  12.3  4.7 |  | **391**  26.1  13.8 | **105**  7.0  3.5 | **213**  14.2  8.8 | **152**  10.1  4.8 | **122**  8.1  4.5 |

**Supplementary Table 2:** Incidence of ADR reports in Germany 2010—2024. Incidence rates (95% confidence interval) per 100,000 dentist years in total and according to medication class.

| **Years** | **Reporting incidence** | **Medication class** |  |  |  |
| --- | --- | --- | --- | --- | --- |
|  | **Total** | **Antibiotics** | **Local anesthetics** | **Analgesics** | **Other medication classes** |
| 2010 | 138.8 (110.8 - 166.9) | 87.1 (64.9 - 109.4) | 19.2 (8.8 - 29.6) | 8.9 (1.8 - 16) | 23.6 (12.1 - 35.2) |
| 2011 | 154.7 (125.3 - 184.2) | 97.8 (74.4 - 121.2) | 26.3 (14.1 - 38.4) | 14.6 (5.6 - 23.6) | 16.1 (6.6 - 25.5) |
| 2012 | 118.4 (92.8 - 144.1) | 76.5 (55.9 - 97.2) | 20.2 (9.6 - 30.8) | 10.1 (2.6 - 17.6) | 11.6 (3.5 - 19.6) |
| 2013 | 104.1 (80.2 - 128) | 59.9 (41.8 - 78) | 20 (9.5 - 30.4) | 12.8 (4.4 - 21.2) | 11.4 (3.5 - 19.3) |
| 2014 | 140.9 (113.3 - 168.4) | 90.2 (68.1 - 112.2) | 21.1 (10.4 - 31.8) | 5.6 (0.1 - 11.2) | 23.9 (12.6 - 35.3) |
| 2015 | 100.5 (77.3 - 123.7) | 67 (48 - 85.9) | 8.4 (1.7 - 15.1) | 2.8 (-1.1 - 6.7) | 22.3 (11.4 - 33.3) |
| 2016 | 141.8 (114.3 - 169.3) | 84.8 (63.5 - 106.1) | 27.8 (15.6 - 40) | 7.0 (0.9 - 13) | 22.2 (11.3 - 33.1) |
| 2017 | 119.2 (94.1 - 144.4) | 86 (64.6 - 107.4) | 11.1 (3.4 - 18.8) | 4.2 (-0.5 - 8.9) | 18 (8.2 - 27.8) |
| 2018 | 99.3 (76.3 - 122.2) | 60.7 (42.7 - 78.6) | 16.5 (7.2 - 25.9) | 5.5 (0.1 - 10.9) | 16.5 (7.2 - 25.9) |
| 2019 | 63.4 (45.1 - 81.7) | 37.2 (23.2 - 51.2) | 12.4 (4.3 - 20.5) | 4.1 (-0.5 - 8.8) | 9.6 (2.5 - 16.8) |
| 2020 | 42.8 (27.7 - 57.8) | 23.5 (12.3 - 34.6) | 4.1 (-0.5 - 8.8) | 5.5 (0.1 - 10.9) | 9.7 (2.5 - 16.8) |
| 2021 | 64.7 (46.2 - 83.1) | 17.9 (8.2 - 27.6) | 11 (3.4 - 18.6) | 4.1 (-0.5 - 8.8) | 31.6 (18.7 - 44.6) |
| 2022 | 50.8 (34.5 - 67.2) | 17.9 (8.2 - 27.6) | 15.1 (6.2 - 24) | 1.4 (-1.3 - 4.1) | 16.5 (7.2 - 25.8) |
| 2023 | 24.7 (13.3 - 36.1) | 8.2 (1.6 - 14.8) | 1.4 (-1.3 - 4.1) | 0 (0 - 0) | 15.1 (6.2 - 24) |
| 2024 | 23.1 (12.1 - 34.1) | 10.9 (3.3 - 18.4) | 2.7 (-1 - 6.5) | 0 (0 - 0) | 9.5 (2.5 - 16.6) |

**Supplementary Table 3:** Incidence of ADR reports in Germany 2010—2024. Incidence rates (95% confidence interval) per 100,000 dentist years in total and according to affected organ system.

| **Years** | **Reporting incidence** | **Organ system** |  |  |  |  |
| --- | --- | --- | --- | --- | --- | --- |
|  | **Total** | **Skin** | **Cardio-vascular system** | **Gastro-intestinal system** | **Central nervous system** | **Other organ system** |
| 2010 | 138.8 (110.8 - 166.9) | 65 (45.8 - 84.2) | 11.8 (3.6 - 20) | 28.1 (15.4 - 40.7) | 16.2 (6.6 - 25.8) | 17.7 (7.7 - 27.8) |
| 2011 | 154.7 (125.3 - 184.2) | 52.6 (35.4 - 69.7) | 19 (8.7 - 29.3) | 39.4 (24.6 - 54.3) | 20.4 (9.7 - 31.1) | 23.4 (11.9 - 34.8) |
| 2012 | 118.4 (92.8 - 144.1) | 57.8 (39.9 - 75.7) | 7.2 (0.9 - 13.6) | 27.4 (15.1 - 39.8) | 15.9 (6.5 - 25.3) | 10.1 (2.6 - 17.6) |
| 2013 | 104.1 (80.2 - 128) | 37.1 (22.8 - 51.3) | 10 (2.6 - 17.4) | 25.7 (13.8 - 37.5) | 21.4 (10.6 - 32.2) | 10 (2.6 - 17.4) |
| 2014 | 140.9 (113.3 - 168.4) | 40.8 (26 - 55.7) | 14.1 (5.4 - 22.8) | 43.7 (28.3 - 59) | 26.8 (14.7 - 38.8) | 15.5 (6.3 - 24.7) |
| 2015 | 100.5 (77.3 - 123.7) | 36.3 (22.3 - 50.2) | 12.6 (4.4 - 20.8) | 22.3 (11.4 - 33.3) | 15.4 (6.3 - 24.4) | 14 (5.3 - 22.6) |
| 2016 | 141.8 (114.3 - 169.3) | 65.3 (46.7 - 84) | 11.1 (3.4 - 18.8) | 26.4 (14.5 - 38.3) | 22.2 (11.3 - 33.1) | 16.7 (7.2 - 26.1) |
| 2017 | 119.2 (94.1 - 144.4) | 55.5 (38.3 - 72.6) | 12.5 (4.3 - 20.6) | 25 (13.4 - 36.5) | 12.5 (4.3 - 20.6) | 13.9 (5.3 - 22.5) |
| 2018 | 99.3 (76.3 - 122.2) | 44.1 (28.8 - 59.4) | 11.0 (3.4 - 18.7) | 22.1 (11.3 - 32.9) | 11 (3.4 - 18.7) | 11 (3.4 - 18.7) |
| 2019 | 63.4 (45.1 - 81.7) | 27.6 (15.5 - 39.6) | 5.5 (0.1 - 10.9) | 12.4 (4.3 - 20.5) | 8.3 (1.7 - 14.9) | 9.6 (2.5 - 16.8) |
| 2020 | 42.8 (27.7 - 57.8) | 23.5 (12.3 - 34.6) | 1.4 (-1.3 - 4.1) | 8.3 (1.7 - 14.9) | 6.9 (0.9 - 12.9) | 2.8 (-1.1 - 6.6) |
| 2021 | 64.7 (46.2 - 83.1) | 12.4 (4.3 - 20.5) | 12.4 (4.3 - 20.5) | 6.9 (0.8 - 12.9) | 15.1 (6.2 - 24.1) | 17.9 (8.2 - 27.6) |
| 2022 | 50.8 (34.5 - 67.2) | 12.4 (4.3 - 20.4) | 13.7 (5.2 - 22.3) | 6.9 (0.8 - 12.9) | 15.1 (6.2 - 24) | 2.7 (-1.1 - 6.6) |
| 2023 | 24.7 (13.3 - 36.1) | 13.7 (5.2 - 22.2) | 1.4 (-1.3 - 4.1) | 1.4 (-1.3 - 4.1) | 2.7 (-1.1 - 6.5) | 5.5 (0.1 - 10.9) |
| 2024 | 23.1 (12.1 - 34.1) | 8.2 (1.6 - 14.7) | 4.1 (-0.5 - 8.7) | 5.4 (0.1 - 10.8) | 4.1 (-0.5 - 8.7) | 1.4 (-1.3 - 4) |

**Supplementary Table 4:** Incidence of ADR reports for different antibiotics in Germany 2010—2024. Incidence rates (95% confidence interval) per 100,000 dentist years according to different antibiotics. *p<0.05

| **Years** | **Reporting incidence** | **Antibiotics** |  |  |  |  |
| --- | --- | --- | --- | --- | --- | --- |
|  | **All antibiotics** | **Clindamycin** | **Amoxicillin** | **Amoxicillin/ Clavulanic acid** | **Metronidazole** | **Others** |
| **Year** |  |  |  |  |  |  |
| 2010 | 87.1 (64.9 - 109.4) | 56.1 (38.3 - 74) | 16.2 (6.6 - 25.8) | 0 (0 - 0) | 10.3 (2.7 - 18) | 11.8 (3.6 - 20) |
| 2011 | 97.8 (74.4 - 121.2) | 74.5 (54 - 94.9) | 42.3 (26.9 - 57.7) | 0 (0 - 0) | 4.4 (0 - 9.3) | 13.1 (4.6 - 21.7) |
| 2012 | 76.5 (55.9 - 97.2) | 39 (24.3 - 53.7) | 26 (14 - 38) | 2.9 (0 - 6.9) | 15.9 (6.5 - 25.3) | 4.3 (0 - 9.2) |
| 2013 | 59.9 (41.8 - 78) | 32.8 (19.4 - 46.2) | 21.4 (10.6 - 32.2) | 1.4 (0 - 4.2) | 4.3 (0 - 9.1) | 5.7 (0.1 - 11.3) |
| 2014 | 90.2 (68.1 - 112.2) | 45.1 (29.5 - 60.7) | 29.6 (16.9 - 42.2) | 5.6 (0.1 - 11.2) | 8.5 (1.7 - 15.2) | 8.5 (1.7 - 15.2) |
| 2015 | 67 (48 - 85.9) | 43.3 (28 - 58.5) | 15.4 (6.3 - 24.4) | 4.2 (0 - 8.9) | 4.2 (0 - 8.9) | 5.6 (0.1 - 11.1) |
| 2016 | 84.8 (63.5 - 106.1) | 26.4 (14.5 - 38.3) | 52.8 (36 - 69.6) | 4.2 (0 - 8.9) | 5.6 (0.1 - 11) | 5.6 (0.1 - 11) |
| 2017 | 86 (64.6 - 107.4) | 34.7 (21.1 - 48.2) | 30.5 (17.8 - 43.2) | 2.8 (0 - 6.6) | 5.5 (0.1 - 11) | 12.5 (4.3 - 20.6) |
| 2018 | 60.7 (42.7 - 78.6) | 22.1 (11.3 - 32.9) | 30.3 (17.7 - 43) | 5.5 (0.1 - 10.9) | 6.9 (0.9 - 12.9) | 1.4 (0 - 4.1) |
| 2019 | 37.2 (23.2 - 51.2) | 13.8 (5.2 - 22.3) | 16.5 (7.2 - 25.9) | 1.4 (0 - 4.1) | 0 (0 - 0) | 9.6 (2.5 - 16.8) |
| 2020 | 23.5 (12.3 - 34.6) | 5.5 (0.1 - 10.9) | 9.7 (2.5 - 16.8) | 4.1 (0 - 8.8) | 2.8 (0 - 6.6) | 2.8 (0 - 6.6) |
| 2021 | 17.9 (8.2 - 27.6) | 2.8 (0 - 6.6) | 9.6 (2.5 - 16.8) | 2.8 (0 - 6.6) | 1.4 (0 - 4.1) | 1.4 (0 - 4.1) |
| 2022 | 17.9 (8.2 - 27.6) | 6.9 (0.8 - 12.9) | 6.9 (0.8 - 12.9) | 2.7 (0 - 6.6) | 0 (0 - 0) | 2.7 (0 - 6.6) |
| 2023 | 8.2 (1.6 - 14.8) | 1.4 (0 - 4.1) | 5.5 (0.1 - 10.9) | 1.4 (0 - 4.1) | 0 (0 - 0) | 0 (0 - 0) |
| 2024 | 10.9 (3.3 - 18.4) | 2.7 (0 - 6.5) | 8.2 (1.6 - 14.7) | 1.4 (0 - 4) | 0 (0 - 0) | 0 (0 - 0) |
